# Particularities of coronary physiology in patients with atrial fibrillation: insights from combined pressure and flow indices measurements

**DOI:** 10.3389/fcvm.2023.1206743

**Published:** 2023-08-14

**Authors:** Georgiana Pintea Bentea, Brahim Berdaoui, Sophie Samyn, Marielle Morissens, Philippe van de Borne, Jose Castro Rodriguez

**Affiliations:** ^1^ Department of Cardiology, CHU Brugmann, Brussels, Belgium; ^2^ Department of Cardiology, CHU Erasme, Brussels, Belgium

**Keywords:** atrial fibrillation, CFR, FFR, HMR, microvascular dysfunction

## Abstract

**Background:**

Symptoms suggestive of myocardial ischemia are frequently encountered in patients with atrial fibrillation (AF) even in the absence of obstructive coronary artery disease. Nevertheless, an in-depth characterisation of coronary physiology in patients with AF is currently lacking.

**Objectives:**

We aim to provide an insight into the characteristics of coronary physiology in AF, by performing simultaneous invasive measurements of coronary flow- and pressure- indices in a real-life population of patients with AF and indication of coronary angiography.

**Methods:**

This is a prospective open label study including patients with permanent or persistent AF and indication of coronary angiography showing intermediate coronary stenosis requiring routine physiological assessment (*n* = 18 vessels from 14 patients). We measured FFR (fractional flow reserve), and Doppler-derived coronary flow indices, including CFR (coronary flow reserve) and HMR (hyperaemic microvascular resistance).

**Results:**

From the analysed vessels, 18/18 vessels (100%) presented a pathological CFR (<2.5), indicative of coronary microvascular dysfunction (CMD), and 3/18 (17%) demonstrated obstructive epicardial coronary disease (FFR ≤ 0.8). A large proportion of vessels (15/18; 83%) showed discordant FFR/CFR with preserved FFR and low CFR. 47% of the coronary arteries in patients with AF and non-obstructive epicardial coronary disease presented structural CMD (HMR ≥ 2.5 mmHg/cm/s), and were associated with high BMR and an impaired response to adenosine. Conversely, vessels from patients with AF and non-obstructive epicardial coronary disease with functional CMD (HMR < 2.5 mmHg/cm/s) showed higher bAPV. The permanent AF subpopulation presented increased values of HMR and BMR compared to persistent AF, while structural CMD was more often associated with persistent symptoms at 3 months, taking into account the limited sample size of our study.

**Conclusion:**

Our findings highlight a systematically impaired CFR in patients with AF even in the absence of obstructive epicardial coronary disease, indicative of CMD. In addition, patients with AF presented more prevalent structural CMD (HMR ≥ 2.5 mmHg/cm/s), characterized by reduced hyperaemic responses to adenosine, possibly interfering with the FFR assessment.

## Background

Atrial fibrillation (AF) is the most common arrhythmia and is associated with significant morbidity driven by heart failure and stroke, and an approximately two-fold increase in premature mortality (1, 2). Around 5%–15% of patients with AF will require coronary stenting (3, 4). However, symptoms suggestive of myocardial ischemia are frequently encountered in patients with AF even in the absence of significant coronary artery disease, that seem to be attributed to myocardial oxygen supply-demand imbalance (5). These could be explained by abnormalities in coronary blood flow associated with AF, which are at the origin of impaired myocardial perfusion. Previous non-invasive studies suggest that patients with AF demonstrate diminished myocardial blood flow reserve and increased coronary vascular resistance indices (6–9). Regarding the invasive assessment of coronary blood flow in patients with AF, data are sparse. A first physiological study using invasive coronary blood flow assessment in subjects with experimentally-induced AF found diminished coronary flow reserve (10). A more recent study showed that patients with AF without significant coronary artery disease had on average lower CFR values, inferring an association of AF with microvascular dysfunction, albeit in the absence of microcirculatory resistance indices measurements (11). Nonetheless, despite these two prior studies, an in-depth characterisation of coronary physiology in patients with AF is currently lacking. As such, we aim to provide an insight into the characteristics of coronary physiology in AF, by performing simultaneous invasive measurements of coronary flow- and pressure- indices in a real-life population of patients with AF and indication of coronary angiography.

## Methods

### Study population

This is a prospective open label study including patients with permanent or persistent AF and indication of coronary angiography (suspected chronic coronary syndrome based on clinical evaluation and non-invasive coronary imaging, or suspected acute coronary syndrome) showing intermediate coronary stenosis (40%–70% visually-assessed stenosis diameter) requiring routine physiological assessment (*n* = 18 vessels from 14 patients). The AF was established to be permanent or persistent in accordance with the 2020 ESC guidelines on AF (12). Patients presented either permanent AF (*n* = 8 patients)—a therapeutic attitude consisting of no additional attempts to restore sinus rhythm, or persistent AF (*n* = 6 patients)—patients who were cardioverted (drugs or electrical cardioversion) usually by the end of the same hospitalization. The exclusion criteria were: cardiac arrest, cardiogenic shock, acute decompensated heart failure, acute phase of ST segment elevation myocardial infarction, culprit vessel in ST segment elevation myocardial infarction and non-ST-segment elevation myocardial infarction, contraindications to adenosine administration (acute asthma, high degree atrioventricular block), and patients in sinus rhythm during the procedure. The prospective study was approved by the Ethical Committee of CHU Brugmann (reference number CE 2020/17). Informed consent was obtained from all patients undergoing investigation.

### Cardiac catheterisation

We measured FFR (fractional flow reserve), and Doppler-derived coronary flow indices—CFR (coronary flow reserve), BSR (basal stenosis resistance), HSR (hyperaemic stenosis resistance), BMR (basal microvascular resistance), and HMR (hyperaemic microvascular resistance) using a ComboWire® (Philips, Volcano) guidewire. Hemodynamic measurements were performed under basal conditions and under hyperaemia induced by intracoronary administration of adenosine (dose of 150 μg, increased progressively until attaining maximal hyperaemia). CFR is calculated as the ratio of maximum blood flow during hyperaemia (hAPV—hyperaemic average peak velocities) and resting coronary blood flow (bAPV—baseline average peak velocities), and reflects both the epicardial and the microcirculatory coronary status (13). Given the irregular RR interval in AF with potential impact on coronary flow, APV was calculated as the average instantaneous peak velocity over an interval of 5 beats. HSR is a combined pressure- and flow-based index defined as the ratio of hyperaemic stenosis pressure gradient (Pa-Pd) and hAPV. A previous study has shown superior diagnostic accuracy of HSR compared to FFR or CFR for assessing the functional significance of epicardial coronary lesions in the general population (13), although is not currently implemented in day-to-day practice. Analogous to HSR, BSR is a combined pressure- and flow-based index that evaluates the epicardial coronary stenosis under basal conditions, being defined as the ratio of resting pressure gradient (Pa-Pd) and bAPV. The microvascular coronary resistances are reflected by BMR under resting conditions (resting Pd/bAPV), and by HMR after adenosine administration (hyperaemic Pd/hAPV) (14).

### Distribution of coronary pressure- and flow-based indices in patients with AF

We assessed the agreement of CFR and FFR by evaluating the concordance of lesion classification obtained using the two techniques. Criteria for revascularization were FFR ≤ 0.8 at maximal hyperaemia (15), and a CFR < 2.5 was considered indicative of coronary microvascular dysfunction (CMD) (16, 17). In addition, we compared FFR to HSR values. HSR higher than 0.8 mmHg/cm/s is associated with significant epicardial coronary artery disease (18).

### Coronary physiological characteristics of patients with AF and non-obstructive epicardial disease

Patients with AF and non-obstructive epicardial coronary disease (FFR > 0.8; 15 vessels from 13 patients) were divided in two subgroups based on the presence of increased microvascular resistance [HMR ≥ 2.5 mmHg/cm/s (19, 20)]. We compared patient and vessel characteristics between the two subgroups (HMR < 2.5 mmHg/cm/s vs. HMR ≥ 2.5 mmHg/cm/s), including differences in pressure- and flow-based indices.

### Clinical findings

In patients with non-obstructive epicardial coronary disease, the coronary physiology indices were compared based on the presence of permanent or persistent AF. In addition, the presence of persistent symptoms was evaluated at 3 months follow-up by the treating cardiologist. The symptoms motivating the coronary angiography were either chest pain or dyspnoea. A systematic differential diagnostic work-up performed prior to the coronary angiography (extensive blood work-up, chest imaging, echocardiography) excluded other potential causes for the presence of the above specified symptoms. Persistent symptoms were defined as the continuous presence of chest pain or dyspnoea at 3 months follow-up. The treating cardiologist was aware of the FFR values, but not of the values of flow-derived coronary indices, at the moment of clinical assessment.

### Statistical analysis

Statistical analyses were performed using GraphPad Prism 9.0.1. Data are presented as mean ± standard deviation. For analysing two groups we employed the Mann-Whitney *U* test. Categorical variables were compared using Chi-square test. Correlation analyses were performed using Spearman's correlation test. The maximum hyperaemic response was analysed using two-way ANOVA, followed by Tukey post-hoc test. Significance level was set at 0.05.

## Results

### Study population

We performed combined assessment of coronary pressure- and flow-derived indices of a total of 18 vessels from 14 patients with AF. The demographic and clinical characteristics of these patients are summarised in Table 1, and of the corresponding vessels in Table 2.

**Table 1:** Characteristics of the study population (*n *= 14 patients).

Age (years)	76.0 ± 9.4
Women/Men (% Women)	5/9 (35.7%)
BMI (kg/m^2^)	25.6 ± 4.8
Cardiovascular risk factors	
– Diabetes Y/N (%Y)	7/7 (50.0%)
– Hypertension Y/N (%Y)	11/3 (78.6%)
– Hypercholesterolemia Y/N (%Y)	10/4 (71.4%)
– Former smoker Y/N (%Y)	6/8 (42.9%)
– Current smoker Y/N (%Y)	2/12 (14.3%)
– Prior ACS Y/N (%Y)	2/12 (14.3%)
– Prior PCI Y/N (%Y)	2/12 (14.3%)
Heart rate (bpm)	81.9 ± 15.7
BP systolic (mmHg)	124.9 ± 23.5
BP diastolic (mmHg)	72.9 ± 12.1
ACS indication Y/N (%Y)	4/10 (28.6%)
LVEF (%)	55.4 ± 10.1
HFpEF Y/N (%Y)	12/2 (85.7%)
HFrEF Y/N (%Y)	2/12 (14.3%)
Cardiac index (L/min/m^2^)	3.4 ± 0.9
Peak NT-proBNP (ng/L)	2880.8 ± 1788.7
Peak Troponin (ng/L)	110.1 ± 135.4
Beta-blockers Y/N	11/3 (78.6%)
RAAS inhibitors Y/N	9/5 (64.3%)

Values are presented as mean ± standard deviation, or group proportions. ACS, acute coronary syndrome; BMI, body mass index; BP, blood pressure; HFpEF, heart failure with preserved ejection fraction; HFrEF, heart failure with reduced ejection fraction; LVEF, left ventricular ejection fraction; PCI, percutaneous coronary intervention; RAAS, renin-angiotensin-aldosterone system. Cardiac index assessed by transthoracic echocardiography.

**Table 2:** Physiological assessment of coronary circulation (*n* = 18 vessels).

	FFR > 0.8	FFR ≤ 0.8
	*n* = 15	*n* = 3
Artery studied		
– LAD/LCX/RCA/Other	8/2/2/3	3/0/0/0
Hemodynamic assessment of epicardial disease		
– FFR	0.89 ± 0.05	0.67 ± 0.07
– HSR (mmHg/cm/s)	0.25 ± 0.20	0.68 ± 0.56
– BSR	0.20 ± 0.20	0.56 ± 0.38
– bAPV (cm/s)	27.5 ± 19.5	46.3 ± 23.7
– hAPV (cm/s)	47.2 ± 30.6	66.0 ± 38.1
– Pa	93.1 ± 14.5	93.3 ± 37.6
– Pd	88.5 ± 14.4	73.3 ± 29.5
– QCA-DS%	53.2 ± 7.6	83.7 ± 8.0
– QCA-length (mm)	14.5 ± 7.1	44.7 ± 7.0
Assessment of the microvasculature		
– CFR	1.74 ± 0.24	1.40 ± 0.17
– HMR (mmHg/cm/s)	2.57 ± 1.34	1.63 ± 1.19
– Increased microvascular resistance (HMR ≥ 2.5 mmHg/cm/s) Y/N (%Y)	7/8 (46.6%)	1/2 (33.3%)
– BMR (mmHg/cm/s)	3.47 ± 1.39	2.73 ± 1.29

Values are presented as mean ± standard deviation, or group proportions. APV average peak velocity; BMR, basal microvascular resistance; BP, blood pressure; BSR, basal stenosis resistance; CFR, coronary flow reserve; DS, diameter stenosis; FFR, fractional flow reserve; HMR, hyperaemic microvascular resistance; HSR, hyperaemic stenosis resistance; LAD, left anterior descending artery; QCA, quantitative coronary angiography.

### Distribution of coronary pressure- and flow-based indices in patients with AF

**Figure 1:**
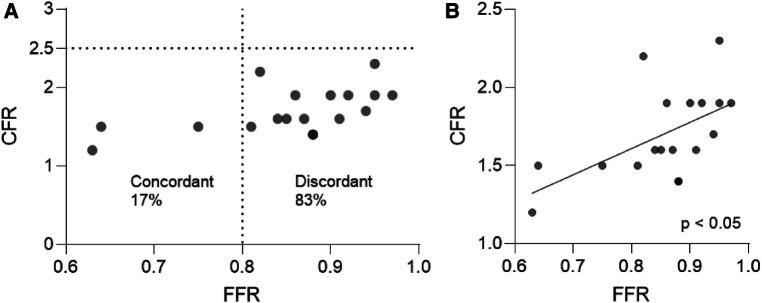
(**A**) Agreement between FFR and CFR indices in patients with AF. (**B**) Correlation analysis between FFR and CFR in patients with AF. Spearman correlation. AF, atrial fibrillation; CFR, coronary flow reserve; FFR, fractional flow reserve.

From the analysed vessels, 18/18 vessels (100%) presented a pathological CFR (<2.5), and only 3/18 (17%) demonstrated obstructive epicardial coronary disease (FFR ≤ 0.8) requiring percutaneous coronary angioplasty. These 3 pathological vessels were distributed in 3 different patients, 2 of which showed one vessel with pathological FFR, and one vessel with non-pathological FFR. As such, most vessels (15/18; 83%) showed discordant FFR/CFR with preserved FFR and low CFR, while 3/18 (17%) showed concordant FFR/CFR with low FFR and low CFR (Figure 1A). We detected a moderate correlation between FFR and CFR (r = 0.5840, *p* = 0.0109; Spearman correlation) (Figure 1B).

### Coronary physiological characteristics of patients with AF and non-obstructive epicardial disease

**Figure 2:**
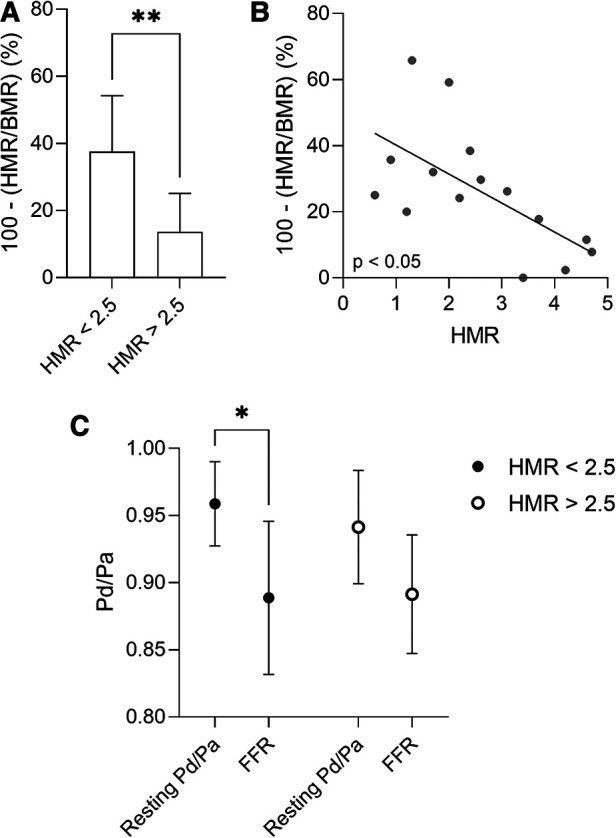
(**A**) Microvascular response to adenosine defined as 100-(HMR/BMR) (%) in patients with AF and non-obstructive epicardial coronary disease, presenting structural (HMR ≥ 2.5 mmHg/cm/s) or functional (HMR < 2.5 mmHg/cm/s) CMD. Data are presented as mean ± standard deviation, and analysed using a Mann-Whitney *U* test, **p < 0.01. (**B**) Correlation between HMR and the microvascular response to adenosine in patients with AF and non-obstructive epicardial disease. Spearman correlation. (**C**) The maximum hyperaemic effect in patients with AF and non-obstructive epicardial disease, presenting structural (HMR ≥ 2.5 mmHg/cm/s) or functional (HMR < 2.5 mmHg/cm/s) CMD. Data are presented as mean ± standard deviation, and analysed using a two-way ANOVA test followed by Tukey's post-hoc test, *p < 0.05. AF, atrial fibrillation; BMR, basal microvascular resistance; CFR, coronary flow reserve; CMD, coronary microvascular dysfunction; FFR, fractional flow reserve; HMR, hyperaemic microvascular resistance.

Vessels of patients with AF and non-obstructive epicardial disease (FFR > 0.8) presented low CFR values (mean value of 1.74 ± 0.24), indicative of CMD. There was no correlation between the CFR values and the left ventricular ejection fraction, or the cardiac index assessed by transthoracic echocardiography (*p* > 0.05; Spearman correlation) as to explain the observed low CFR values. Using the described cut-off of 2.5 mmHg/cm/s for pathological HMR (19, 20), and the pattern of structural/functional CMD identified in the general population (16), we divided the vessels of patients with AF and non-obstructive epicardial disease as presenting structural CMD (HMR ≥ 2.5 mmHg/cm/s; 7 coronary arteries and 6 patients) or functional CMD (HMR < 2.5 mmHg/cm/s; 8 coronary arteries and 7 patients) (Tables 3, 4). The subgroup of vessels with structural CMD demonstrated significantly higher BMR values compared to the vessels with functional CMD (4.34 ± 0.67 mmHg/cm/s vs. 2.71 ± 1.43 mmHg/cm/s; *p* = 0.0289) (Table 4). High HMR was associated with a reduced microvascular response to adenosine defined as 100-(HMR/BMR) (%) when compared to low HMR (13.6 ± 11.4% vs. 37.5 ± 16.7%; *p* = 0.0093) (Figure 2A). In addition, there was a moderate inverse correlation between HMR values and the microvascular response to adenosine (Figure 2B) (r = −0.6429, *p* = 0.0116; Spearman correlation). In line with these findings, the maximum hyperaemic effect in vessels of patients with non-obstructive epicardial disease was found to depend on the presence of increased microvascular resistance, as adenosine administration led to a significant decrease in Pd/Pa only in vessels with HMR < 2.5 mmHg/cm/s (Figure 2C). A more detailed look at the clinical and lesion characteristics of patients with non-obstructive epicardial disease according to the presence of increased microvascular resistance is overviewed in Tables 3, 4. The subgroup with functional CMD presented higher bAPV (36.4 ± 23.4 cm/s vs. 17.4 ± 4.9 cm/s; *p* = 0.0294) and hAPV (62.4 ± 34.8 cm/s vs. 29.8 ± 10.7 cm/s; *p* = 0.0347) compared to the subgroup with structural CMD.

**Table 3:** Characteristics of patients with AF and non-obstructive epicardial coronary disease, presenting structural (HMR ≥ 2.5 mmHg/cm/s) or functional (HMR < 2.5 mmHg/cm/s) CMD (*n* = 13 patients).

	Functional CMD (HMR < 2.5 mmHg/cm/s)	Structural CMD (HMR ≥ 2.5 mmHg/cm/s)	*P*
	*n* = 7	*n* = 6	
Age (years)	74.6 ± 11.7	78.9 ± 6.5	0.836
Women/Men	2/5	3/3	0.428
Diabetes Y/N	3/4	3/3	0.796
Hypertension Y/N	5/2	5/1	0.611
Hypercholesterolemia Y/N	5/2	4/2	0.852
Kidney failure Y/N	2/5	2/4	0.852
LVEF (%)	54.0 ± 9.4	57.0 ± 12.2	0.509
HFpEF Y/N	6/1	5/1	0.905
HFrEF Y/N	1/6	1/5	0.905
Cardiac index (L/min/m^2^)	3.08 ± 0.78	3.54 ± 0.91	0.484
LV filling pressure Normal/High	3/4	3/2	0.558
Peak NT-proBNP (ng/L)	2865.2 ± 1743.8	3533.3 ± 2075.9	0.786
Peak Troponin (ng/L)	161.4 ± 168.1	54.6 ± 46.9	0.171
Chest pain Y/N	4/3	2/4	0.390
Dyspnoea Y/N	3/4	4/2	0.390
ACS indication Y/N	3/4	1/5	0.307
Positive NCI Y/N	5/2	4/2	0.852
3-mo persistent symptoms Y/N	0/7	5/1	<0.01
Permanent/Persistent AF	2/5	6/0	<0.01
Beta-blockers Y/N	6/1	4/2	0.416
RAAS inhibitors Y/N	5/2	3/3	0.428
Heart rate (bpm)	76.7 ± 14.8	81.7 ± 6.7	0.558
Heart rate Beat-to-beat variability (CV%)	0.12 ± 0.05	0.12 ± 0.04	0.947

Values are presented as mean ± standard deviation, or group proportions. *P* values were determined using Mann-Whitney *U* test, or Chi-square test respectively. ACS, acute coronary syndrome; AF, atrial fibrillation; CMD, coronary microvascular dysfunction; HFpEF, heart failure with preserved ejection fraction; HFrEF, heart failure with reduced ejection fraction; LVEF, left ventricular ejection fraction; NCI, non-invasive coronary imaging; QCA, quantitative coronary angiography; RAAS, renin-angiotensin-aldosterone system. Cardiac index assessed by transthoracic echocardiography.

**Table 4:** Characteristics of vessels with AF and non-obstructive epicardial coronary disease, presenting structural (HMR ≥ 2.5 mmHg/cm/s) or functional (HMR < 2.5 mmHg/cm/s) CMD (*n* = 15 vessels).

	Functional CMD (HMR < 2.5 mmHg/cm/s)	Structural CMD (HMR ≥ 2.5 mmHg/cm/s)	*P*
	*n* = 8	*n* = 7	
LAD/LCX/RCA/Other	4/1/1/2	4/1/1/1	0.966
QCA-DS%	51.6 ± 8.3	55.0 ± .8	0.749
QCA-length (mm)	14.7 ± 6.9	14.3 ± 7.9	0.942
Resting Pd/Pa	0.96 ± 0.03	0.94 ± 0.04	0.927
FFR	0.89 ± 0.06	0.89 ± 0.04	0.976
CFR	1.80 ± 0.26	1.67 ± 0.23	0.391
HSR (mmHg/cm/s)	0.16 ± 0.14	0.35 ± 0.21	0.077
HMR (mmHg/cm/s)	1.54 ± 0.64	3.76 ± 0.78	<0.001
BMR (mmHg/cm/s)	2.71 ± 1.43	4.34 ± 0.67	<0.05
BSR	0.12 ± 0.09	0.27 ± 0.26	0.304
HMR/(HMR + HSR)	0.91 ± 0.06	0.92 ± 0.04	>0.99
Microcirculatory resistance response to adenosine 100—(HMR/BMR) (%)	37.5 ± 16.7	13.6 ± 11.4	<0.01
bAPV (cm/s)	36.4 ± 23.4	17.4 ± 4.9	<0.05
hAPV (cm/s)	62.4 ± 34.8	29.8 ± 10.7	<0.05

Values are presented as mean ± standard deviation, or group proportions. *P* values were determined using Mann-Whitney *U* test, or Chi-square test respectively. bAPV baseline average peak velocity; BMR, basal microvascular resistance; BSR, basal stenosis resistance; CFR, coronary flow reserve; CMD, coronary microvascular dysfunction; DS, diameter stenosis; FFR, fractional flow reserve; hAPV, hyperaemic average peak velocity; HMR, hyperaemic microvascular resistance; HSR, hyperaemic stenosis resistance; LAD, left anterior descending artery; QCA, quantitative coronary angiography.

### Clinical findings

**Figure 3:**
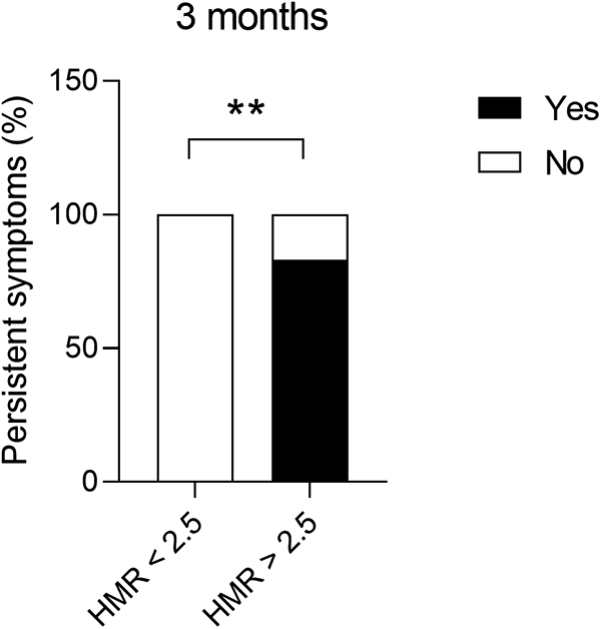
Presence of persistent symptoms (chest pain or dyspnoea) at 3 months following invasive assessment in patients with AF and non-obstructive epicardial coronary disease, presenting structural (HMR ≥ 2.5 mmHg/cm/s) or functional (HMR < 2.5 mmHg/cm/s) CMD. Chi-square test, **p < 0.01. AF, atrial fibrillation; CMD, coronary microvascular dysfunction; HMR, hyperaemic microvascular resistance.

**Figure 4:**
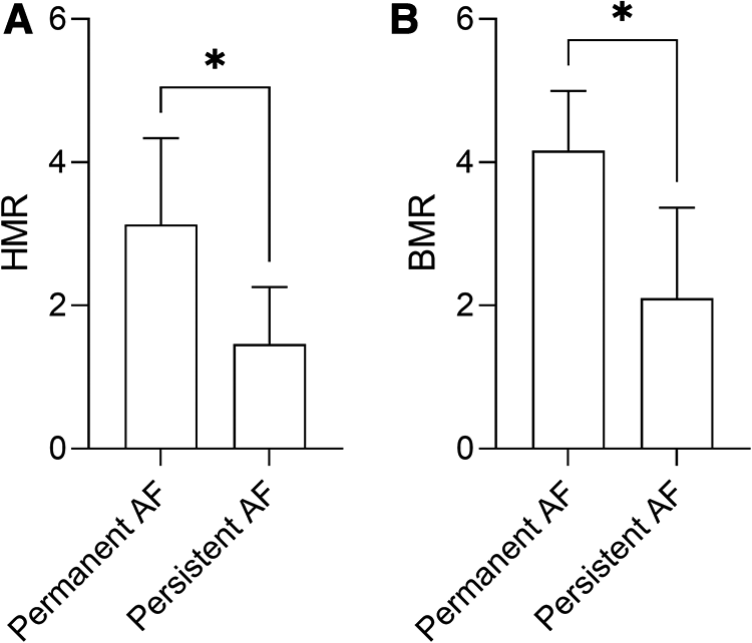
HMR (**A**) and BMR (**B**) in patients with permanent or persistent AF, and non-obstructive epicardial disease. Data are presented as mean ± standard deviation, and analysed using a Mann-Whitney *U* test, *p < 0.05. AF, atrial fibrillation; BMR, basal microvascular resistance; HMR, hyperaemic microvascular resistance.

Patients with structural CMD demonstrated significantly more persistent symptoms (chest pain or dyspnoea) at 3 months following invasive assessment (83.3% vs. 0.00%, *p* = 0.0021) compared to functional CMD (Figure 3). Furthermore, permanent AF was more often associated with high HMR (3.13 ± 1.20 mmHg/cm/s vs. 1.46 ± 0.79 mmHg/cm/s, *p* = 0.0193) (Figure 4A) and high BMR (4.16 ± 0.83 mmHg/cm/s vs. 2.10 ± 1.26 mmHg/cm/s, *p* = 0.0127) (Figure 4B) compared to persistent AF.

**Figure 5:**
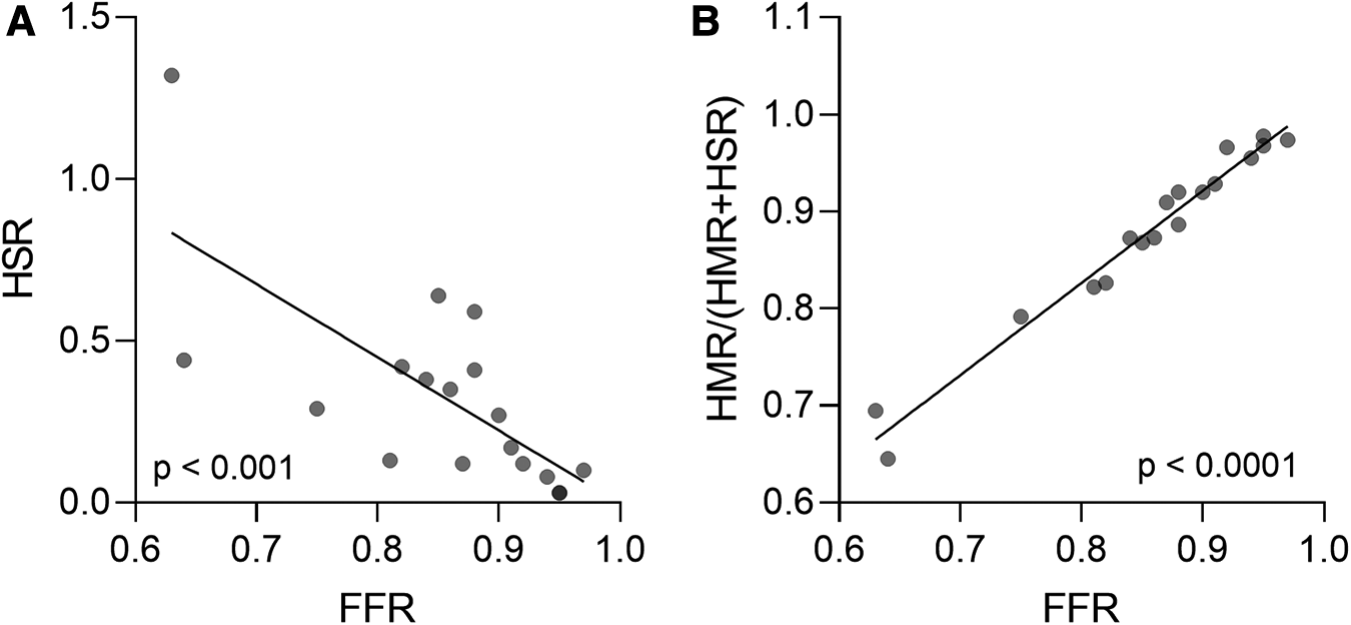
(**A**) Agreement between FFR and HSR in evaluating coronary stenosis severity in patients with AF. (**B**) Correlation between FFR values and the microvascular component of total vascular resistance, defined as HMR/(HMR + HSR) in patients with AF. Spearman correlation. AF, atrial fibrillation; FFR, fractional flow reserve; HMR, hyperaemic microvascular resistance; HSR, hyperaemic stenosis resistance.

Regarding the functional evaluation of coronary stenosis severity, there was high agreement in the classification of the lesions between FFR and HSR (83%), with the two indices showing strong correlation across all patients with AF (r = −0.7446, *p* = 0.0004; Spearman correlation) (Figure 5A). Moreover, FFR values correlated strongly with the microvascular component of total vascular resistance, defined as HMR/(HMR + HSR) (r = 0.9866, *p* < 0.0001; Spearman correlation) (Figure 5B).

## Discussion

In the present study, our main findings indicate that: (1) all patients with AF have impaired CFR (<2.5) even in the absence of obstructive epicardial coronary disease, indicative of CMD; (2) a large proportion of the studied vessels (83%) showed discordant FFR/CFR with preserved FFR and low CFR; (3) 47% of the coronary arteries in patients with AF and non-obstructive epicardial coronary disease presented structural CMD defined by high HMR (≥2.5 mmHg/cm/s), with a correspondingly high BMR and an impaired response to adenosine, possibly interfering with the FFR assessment; conversely, vessels from patients with AF and non-obstructive epicardial coronary disease with functional CMD (HMR < 2.5 mmHg/cm/s) showed higher bAPV, which could explain the reduced CFR in this subpopulation; (4) the permanent AF subpopulation presented increased values of HMR and BMR compared to persistent AF, while high HMR (≥2.5 mmHg/cm/s) was more often associated with persistent symptoms at 3 months, taking into account the limited sample size of our study.

### CFR in patients with AF

All patients with AF in our study presented with diminished CFR (<2.5), even in the absence of obstructive coronary artery disease, indicative of CMD. There are multiple studies showing that myocardial ischemia follows more closely changes in coronary blood flow than coronary perfusion pressure (21, 22). Furthermore, low CFR values (<2.5), as observed in the current study, were associated with an excess of major adverse cardiovascular events (MACE) and target vessel failure at 5-year follow-up (17). Our findings of abnormal CFR in patients with AF are consistent with previous reports of decreased hyperaemic myocardial blood flow in AF assessed by non-invasive methods (6–9). In addition, Kochiadakis et al. demonstrated diminished CFR in acute experimentally-induced AF in subjects with normal angiographic coronary arteries by means of invasive coronary physiology assessment (10). This has been postulated to occur due to the irregular ventricular rhythm associated with AF. It took close to twenty years after this initial investigation for a more recent study to revisit the evaluation of CFR in AF using invasive coronary assessment. In this study, Ozcan et al. showed lower CFR values in patients with AF and non-obstructive coronary artery disease, hypothesised to second the presence of microvascular dysfunction, however without evaluating specifically the microcirculation (11). The observed low CFR in these patients could be partly due to the highly prevalent association of AF with heart failure (23), or it could be a purveyor of arrhythmia-induced cardiomyopathy. Furthermore, we observed a mean value of CFR of 1.74 in patients with AF and non-obstructive coronary artery disease, which is similar to that observed by Ozcan et al. (1.70) (11), however lower than that obtained by Kochiadakis et al. (2.15) (10). Although all three studies suggest the presence of CMD in AF as assessed by CFR (<2.5), the higher mean values obtained in the latter study could be explained by CFR measurements performed in acutely-induced AF (10).

### Distribution of coronary pressure- and flow-based indices in patients with AF

Interestingly, we could observe a highly prevalent discordance between CFR and FFR (FFR > 0.8 and CFR < 2.5) in 83% of patients. In the general population, this particular discordance of FFR/CFR was observed in approximately 25% of patients according to recent studies (24, 25), although a previous study reported this discordant population in as low as 6.4% of cases (26). Patients with discordant FFR/CFR presented higher MACE rates than the concordant normal FFR/CFR group at 1 year (27) and 10 years (26) follow-up. Nonetheless, regardless of the high rate of discordance of FFR/CFR in terms of their established cut-offs, we could demonstrate a significant, albeit moderate correlation between the absolute FFR and CFR values in patients with AF, similar as reported in the general population (28).

HSR was found to have superior diagnostic accuracy compared to FFR and CFR when confronted to non-invasive imaging using single photon emission computed tomography (18), and has been used for evaluating the accuracy of pressure-based indices in the general population (29, 30). In the current study, we observed that 83% of patients showed a concordance of FFR and HSR, using a cut-off for HSR of 0.8 mmHg/cm (18). This is consistent with the previously reported diagnostic agreement between FFR and HSR in the general population of 78.7% (30). However, HSR is also dependent on an adequate hyperaemic response, and can be confounded by the presence of microvascular dysfunction (13). Previously, Kochiadakis et al. indicated that irregularities in ventricular rhythm may be responsible of diminished CFR in AF (10), while a recent case report by Mills et al. highlighted that changes in cardiac rhythm associated with AF may impact coronary physiological assessment (31). In a retrospective setting, we observed that FFR was more robust than instantaneous wave-free ratio (iFR), a resting coronary pressure index, in evaluating coronary lesions in AF, showing similar variability, reproducibility, and lesion classification as observed in patients with sinus rhythm (32). In addition, adenosine-induced hyperaemia during FFR measurement was found to be equivalent in AF compared to sinus rhythm in terms of magnitude and duration (33). On the other hand, iFR presented poorer reproducibility in lesion classification in AF, and was prone to increased beat-to-beat variability which correlated with the heart rate variability (32), supporting the notion that abnormalities in cardiac rhythm may impact evaluation of coronary physiology using resting physiological indices. Further studies are necessary to provide a better understanding on the impact of cardiac rhythm on coronary physiological assessment in AF.

### Microvascular dysfunction in AF

The low CFR observed in patients with AF and non-obstructive epicardial coronary disease indicates the presence of CMD in this population (34). CMD is a result of functional or structural alterations of the endothelium (35). Mechanistically several pathogenic hypotheses were described including impaired NO-mediated vasodilatation, as a consequence of glycocalyx degradation due to oxidative stress, inflammation or ischemia (35), triggers which have been described in AF (36). In addition, CMD was also hypothesized to occur secondary to alterations in coronary flow distribution due to compromised intercellular signal transmission, or to impaired metabolic signalling (35). Furthermore, structural modification of the microcirculation such as capillary rarefaction and remodelling can contribute to increased coronary microvascular resistance (37). Previous studies have indicated the presence of microvascular coronary dysfunction in AF (11). Using positron emission tomography, Range et al. identified increased coronary vascular resistance under hyperaemic conditions in patients with AF (9). Similarly, Kochiadakis et al. observed increased hyperaemic coronary vascular resistance in subjects with acute experimentally-induced AF compared to sinus rhythm using invasive coronary assessment (10). The microvascular dysfunction observed in AF could be responsible for the ischemic manifestation (chest pain, increased troponin, ECG modifications) in the absence of significant epicardial coronary disease (38). In addition, the presence of microvascular disease in the general population was shown to be associated with poorer prognosis in terms of cardiac mortality (39).

The microvascular coronary status is reflected in terms of coronary indices by HMR and BMR. Recent studies have identified that HMR values above 2.5 mmHg/cm/s are suggestive of increased microvascular resistance (19, 20) defining structural CMD, whereas functional CMD is characterized by HMR < 2.5 mmHg/cm/s in presence of diminished CFR (16, 40). Interestingly, we identified amongst the population with AF and non-obstructive epicardial coronary disease, 47% (7/15) vessels showing HMR ≥ 2.5 mmHg/cm/s, which contrasts to approximately 21% reported in the general population (16). Using this threshold, we divided the patients with AF and non-obstructive epicardial disease as presenting structural CMD (HMR ≥ 2.5 mmHg/cm/s) or functional CMD (HMR < 2.5 mmHg/cm/s), demonstrating different physiological and clinical characteristics. Coronary arteries from patients with AF and structural CMD showed higher BMR values, impaired response to adenosine, lower bAPV and hAPV, and were more frequently associated with permanent AF.

High HMR values were described as a predictor of myocardial ischemia even in the absence of significant epicardial coronary lesions (14). In addition, high HMR values could influence the evaluation of coronary physiology using pressure-based indices (41, 42). Specifically in the AF population, a retrospective study found that iFR was less reproducible in assessing intermediate coronary stenoses (32), that could potentially be explained by the increased microvascular resistance. In addition, in the current study we have observed a strong correlation between FFR and the ratio between HMR and the total vascular resistance (HMR + HSR). Previously, we have reported similar hyperaemic responses to adenosine in an overall retrospective population of patients with AF compared to sinus rhythm, however without having available their microvascular resistance characteristics (33). In the present study, we showed that vessels from patients with AF and high HMR have a reduced hyperaemic response compared to the subgroup of AF and low HMR. Furthermore, we demonstrated that HMR values were inversely correlated with the magnitude of the response to adenosine. This aspect should be considered when assessing epicardial coronary lesion severity using FFR in patients with AF and structural CMD.

Patients with AF and functional CMD present significantly higher bAPV, which could explain the low CFR in this subgroup even in the presence of normal microvascular resistance, as there is a maximum reachable hyperaemia (43). Previous observations indicate that acute experimentally-induced AF leads to increases in baseline coronary blood flow, that could be partly explained by an increase in heart rate, and hypothesised to be the result of coronary vasodilation secondary to higher metabolic demands in AF (10). This is in line with our findings that patients with higher bAPV present lower BMR. Another potential explanation of high bAPV reported in the literature was the dysfunction of coronary autoregulation which was associated with adverse long-term outcomes (39). With increasing AF burden, endothelial dysfunction, sympathetic activation, progressive left ventricular fibrosis and both atrial and ventricular remodelling were described, that could impact irreversibly the coronary microcirculation (36). Accordingly, patients with AF and high HMR showed increased BMR and impaired response to adenosine, potentially preventing them from reaching maximum hyperaemia.

### Clinical findings

In our study at 3 months follow-up, patients with AF and non-obstructive epicardial coronary disease presenting with structural CMD demonstrated higher percentage of persistent symptoms, compared to patients with functional CMD. It should be noted that persistent symptoms were solely defined as present or absent, without providing specific quantification of symptom burden. It has been previously hypothesised that the increased microvascular resistance could be responsible for the symptoms suggestive of myocardial ischemia in AF even in the absence of significant coronary artery disease (36). Our findings seem to support this hypothesis, as we observed more persistent symptoms in patients with AF and increased microvascular resistance in the absence of obstructive coronary artery disease as assessed by FFR. Nevertheless, given the higher prevalence of persistent symptoms in the subpopulation of AF with increased HMR, and the strong correlation between FFR and the microvascular component of the total vascular resistance, possibly a part of the FFR/CFR discordance observed in patients with AF is related to an underestimation of coronary artery stenosis assessed by FFR. Indeed, in our study, solely 17% (3/18) vessels were found to have pathologic FFR (≤0.8), and thus requiring revascularization, which is in contrast with the general population estimates of about 45%–50% (44, 45). Future studies will be important to address this hypothesis. Interestingly, performing coronary angioplasty in cases of FFR negative lesions, but showing high HMR, has been described in literature as an efficient rescue therapy in case of persistent symptoms (42). Alternatively, the difference in clinical outcome may also be explained by the presence of predominantly permanent AF in the subgroup with structural CMD, and more prevalent persistent AF in the subgroup with functional CMD. Notably, only 2 out of the 7 patients with functional CMD and absence of persistent symptoms were in sinus rhythm at 3 months clinical follow-up.

Furthermore, as indicated above, it is the subpopulation of permanent AF which presents increased HMR and BMR compared to persistent AF. As such, particular attention is warranted clinically in interpreting the coronary pressure-based indices in this subgroup. Interestingly, in this study, 7 out of the 8 patients with permanent AF experienced AF for a duration of at least 1 year, while 4 out of the 6 patients with persistent AF presented *de novo* AF. This difference in the duration of AF between the two groups could perhaps impact the observed changes in microcirculation characteristics. This could mirror the observed bimodal pattern of coronary microvascular dysfunction (functional CMD, followed by structural CMD) described in diabetes mellitus in a time-dependent manner (40), and more recently in the general population (16).

### Study limitations

We acknowledge that the present study has limitations, including the small sample size, especially regarding the clinical findings, driven by the current shortage of commercial ComboWire® guidewires, the lack of a control group in sinus rhythm, and the limited clinical follow-up, as it was designed firstly as a physiological single-centre study. In addition, the clinical evaluation included only the presence or absence of persistent symptoms, without providing specific quantification of symptom burden. Furthermore, our observations cannot be extrapolated to patients with paroxysmal AF, as they were not included in the study population.

## Conclusion

This is the first study characterising the coronary physiology in a real-life AF population using simultaneous assessment of pressure- and coronary flow- indices. We demonstrate that all studied vessels from patients with AF and non-obstructive epicardial coronary disease have impaired CFR (<2.5) indicative of CMD, and we identified an increased prevalence of structural CMD (HMR ≥ 2.5 mmHg/cm/s) compared to the general population. Vessels from patients with AF and structural CMD showed reduced hyperaemic responses to adenosine, possibly interfering with the FFR assessment. In addition, we found that permanent AF presented increased HMR and BMR compared to persistent AF.

## Data Availability

The raw data supporting the conclusions of this article will be made available by the authors, without undue reservation.
